# Social Cognitive Orientations, Social Support, and Physical Activity among at-Risk Urban Children: Insights from a Structural Equation Model

**DOI:** 10.3390/ijerph17186745

**Published:** 2020-09-16

**Authors:** Junghyae Lee, John Hoornbeek, Namkyung Oh

**Affiliations:** 1 Begun Center for Violence Prevention Research and Education, Jack, Joseph and Morton Mandel School of Applied Social Sciences, Case Western Reserve University, Cleveland, OH 44106, USA; 2 Center for Public Policy and Health, College of Public Health, Kent State University, Kent, OH 44242, USA; jhoornbe@kent.edu; 3 School of Public Administration, University of Nebraska Omaha, Omaha, NE 68182, USA; noh@unomaha.edu

**Keywords:** physical activity, urban children, structural equation modeling, social cognitive theory, parental support, peer support, teacher support, self-efficacy, exercise enjoyment, behavioral intention, school children, pupils

## Abstract

This study investigates the effects of cognitive orientations associated with social cognitive theory (SCT) and exercise enjoyment on physical activity (PA) of urban at-risk children, accounting for mediating effects associated with various sources of social support. We use 2016–2017 survey data from 725 school-age children in an urban school district in Akron, Ohio in the United States (US) to inform a structural equation model, which assesses direct and indirect effects of self-efficacy, behavioral intention, and exercise enjoyment on children’s PA, using mediating variables that measure social support that children report receiving from parents, Physical Education (PE) teachers, and peers. We find that self-efficacy and exercise enjoyment have notable direct and indirect effects on the children’s PA. We also find that the support children receive from PE teachers and peers appears to have greater effects on PA than does the children’s reported social support from parents. These findings suggest that children’s social cognitive orientations may influence both sources of perceived social support and the extent to which children engage in PA. While these findings have potential implications for intervention strategies to increase PA among at-risk children, further research is appropriate to improve our understanding of the determinants of PA among at-risk urban children.

## 1. Introduction

Regular physical activity (PA) can prevent certain diseases, which, in turn, can reduce healthcare costs [1,2,3,4]. National data on children’s health in the United States (US) show that racial/ethnic minority and lower-income children are less likely to participate in PA [5,6,7]. This lower engagement in PA can increase children’s chances of adverse health outcomes such as obesity, diabetes, cardiovascular disease, and hypertension [8,9]. Research has documented that children’s PA varies by ethnicity [10], and self-reported PA for children aged 9 to 13 suggests that African American adolescents participate in less PA than their peers [10,11]. In addition, children aged 5 to 10 years in low-income families show lags in behavioral development and health compared to their peers in higher-income families [12]. For all of these reasons, there is a need to identify factors affecting the PA of low income and racial/ethnic minority children. While the concept of PA can be distinguished from the concept of “exercise”, which tends to involve more planning than is implied by the term PA [13,14], children’s reactions during pilot testing of the survey instrument used to collect data for this study suggest that this distinction is not recognized easily by the young children who are the subjects in this study. We therefore used the terms interchangeably both with the children surveyed and in the narrative of this article.

A key psychological theory used to understand human behavior is social cognitive theory (SCT), and it has been developed and applied to health promotion by Dr. Albert Bandura [15]. In general, it suggests that there are reciprocal relationships between environmental and cognitive factors that affect individual behavior [16,17]. It also envisions two key cognitive constructs—self-efficacy and behavioral intentions relating to specific outcomes—playing key roles in driving individual behaviors, including those relating to PA. Self-efficacy is a belief in one’s abilities to deal with various situations [14,17]. This belief can play an important role in how one manages their health. Behavioral intention relates to one’s expectations relating to the results of performing a certain behavior [15]. In Bandura’s view, these cognitive factors—in combination with environmental factors—function as a trigger for behavioral decisions relating to PA engagement [15,18].

While there is evidence that psycho-cognitive factors implicit in SCT influence PA [19,20,21,22,23], there is also evidence suggesting that exercise enjoyment helps facilitate PA in children. Simpson and colleagues found that the promotion of exercise enjoyment encourages higher levels of PA, particularly among children with low self-efficacy [24]. Barr-Anderson and her colleagues found that among black children, PA was positively associated with children’s enjoyment of physical education (PE) and parental support for PA, based on information reported by parents [25].

There is also reason to believe that childhood PA may be affected by environmental factors, including social support from influential people in children’s lives [2,26,27,28,29,30,31]. The impact of parents, PE teachers, and peers has been investigated and substantiated in past studies [26,31,32,33]. For example, Chen et al. [33] found that peer-support is an important predictor of PA, particularly in middle school and younger children. However, there remains a need to further clarify the impacts of influential types of social support on childhood PA [34,35]—particularly for children in low income and racial/ethnic minorities. Lee et al.’s recent study [36] takes steps in this area, as it found that school PE teachers’ motivational styles affect PA outside the classroom. However, Lee and colleagues’ study [36] does not account for student perceptions of parental and peer support nor does it account for cognitive orientations that may influence children’s perceived support from PE teachers, parents, and/or peers. As a result, it did not analyze social support variables as thoroughly as it might and in ways that recognize the potential prior influence of social-cognitive factors [17,20].

In this study, we seek to expand our knowledge in this area by addressing two research questions:

1. What are the direct, indirect and total effects of key cognitive orientations (self-efficacy, exercise enjoyment, and behavioral intention) on the PA of children in at-risk low-income neighborhoods?
2. What are the impacts of parental, PE teacher, and peer support on children’s PA in at-risk low-income neighborhoods?

To address these questions, we use cognitive orientations drawn from SCT and other previous work [24] to build upon a previously published study [36] on the impact of specific forms of social support on PA practices of low income ethnic/racial minority children in an urban area of a midwestern city in the United States (US). In doing so, we also draw on previous work on cognitive orientation that suggests that cognitive processes orient individual attention and focus toward external stimuli in ways that affect both the meaning of those stimuli and individual health behavior [37,38]. Our approach is also informed by Bandura’s work [15,16,17,18,39], suggesting that cognitive orientations of individuals and environmental factors such as social support operate reciprocally and may influence health promotion activities, as is mentioned briefly above. Specifically, we seek to assess how cognitive orientations (as measured by specific “correlates”) associated with self-efficacy, behavioral intention, and exercise enjoyment influence both children’s perceived social support from peers, parents, and PE teachers and their PA.

We use structural equation modeling (SEM) to assess the direct and indirect impacts of theoretically important social psychological factors on PA, while also supplementing and strengthening understandings associated with Lee et al.’s [36] recently published study of the impacts of social support structures on children from low income ethnic/racial minority families. In doing so, we offer further—and arguably more complete—evidence on the impact of theoretically derived variables drawn from previous work [17,19,20,40], as well as enhanced insights regarding factors that may influence both PA and the long term health prospects for children from low income and ethnic/racial minority families. Our hope and expectation is that these insights will productively inform future interventions and research in this area.

## 2. Materials and Methods

Our analyses are based on survey data and structural equation modeling (SEM), which were used to analyze the influence of cognitive orientations associated with self-efficacy, behavioral intention, and exercise enjoyment on perceived sources of social support and reported PA of children in our sample. In the subsections that follow, we outline the conceptual relationships underlying the SEM model, the sources of data used and the manner in which they were collected, the measures of key concepts used in the model, and the statistical analyses conducted.

### 2.1. The Conceptual Model

Bandura suggests that a belief in one’s self-efficacy is important to one’s motivation, behavior, and accomplishments [15,16,39]. He also suggests that cognitive orientations and environmental influences (including social support) may influence each other in reciprocal fashion, such that social supports may influence cognitive orientations and vice versa [16]. While past research has analyzed the influence of social support on cognitive orientations (see Rovniak et al., 2002 [40], for example), we focus on potential ways in which cognitive orientations may influence both perceived social support from differing sources and PA. In so doing, we recognize that children may assign meaning to differing sources of social support differently, depending on the nature and strength of their cognitive orientations.

**Figure 1:**
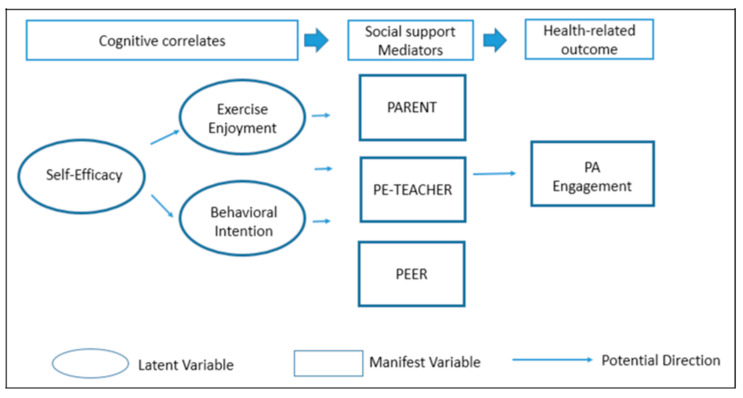
Schematic illustration of the conceptual model.

The conceptual model used to guide the SEM model used in this work is outlined in Figure 1. Due to their central role in motivating individual and health-related behaviors [15,16,17,18,39], the model posits that feelings of self-efficacy may influence both children’s behavioral intentions and their enjoyment of exercise. The model also examines relations between self-efficacy and other cognitive orientations (measured as latent “correlates”) and the mediating effects of varying sources of social support on PA. In doing so, we assess children’s cognitive correlates as potential factors influencing both their perceived sources of social support and their PA. In this regard, the cognitive correlates we analyze include behavioral intention and exercise enjoyment, and we examine their influence on parental, PE teacher, and peer support, as well as the reported PA of the children surveyed. The results of our analysis include estimates of the direct and indirect effects of the cognitive correlates on PA, as well as estimates of the impacts of various forms of social support on PA reported by children in our sample.

### 2.2. Data Sources and Collection: Participant Characteristics and Recruitment

We collected data from surveys of school children conducted at four Akron public schools within the Akron city school district in the US, where school students were largely from low-income families served by the federal free school lunch program [41]. This program provides in-school meals for children in households with incomes at or below 130 percent of the federal poverty level in the US, under the United States Department of Agriculture (USDA) community eligibility program. Among the participating schools, any child enrolled in grades 2–5 (mainly aged 5 to 10 years) during the 2016–2017 school year was eligible to participate in the study, with the exception of those with physical disabilities that prevented their participation or those with mental impairment that hampered their abilities to understand the survey questionnaire, which includes children’s sex, race, grade, family type, and cognitive orientation (self-efficacy, behavioral intention, exercise enjoyment), social support (parent, PE teacher, and peer), and PA measure.

In total, 1325 consent forms were distributed to students during PE classes, but 513 students did not return consent forms and 68 students refused to participate (response rate = 56.2%). As a result, we received survey responses from 744 students, and 725 of these surveys were sufficiently complete to include in the analysis. Children who participated in the study were asked to complete the survey questionnaire in school recess and lunch hour. The Kent State University Institutional Review Board (IRB) and the Akron city schools district board gave approval for the study, and consent was obtained from both the caregivers for the children surveyed and the participating children.

### 2.3. Measures

Data from the survey were collected to create measures of PA, cognitive correlates reflecting key cognitive orientations (e.g., constructs) for the children in the sample, and social support the children reported receiving from parents, PE teachers and peers. We briefly overview measures used in each of these areas below.

#### 2.3.1. Measure of Physical Activity (PA)

The PA outcome variable is a composite measure that was compiled from information provided in self-administered responses from students. The question(s) were derived from the Global Physical Activity Questionnaire (GPAC), which is comprised of 16 items designed to assess the frequency and duration of PA during week [42]. The items assessed include a range of different kinds of physical activity in which children may engage. The GPAQ has established reliability and validity characteristics, and the questions it uses have been used in other PA monitoring studies [43,44,45].

The GPAQ enables children to report the total amount of time they spent on PA over 7 consecutive days to obtain a measure of habitual PA. The measure asks specifically about 16 components of PA, including softball, basketball, walking, baseball, and multiple other forms of PA [42]. Using responses received in the survey questionnaire, we added time reported across all of these activities to arrive at an aggregated estimate of total PA over the last 7 consecutive days. More specifically, the daily time spent in PA is calculated by summing the minutes the student reports spending on each of these activities and then summing the totals for each activity to create a measure of total PA time during the last 7 days [46].

#### 2.3.2. Cognitive Constructs (Self-Efficacy, Behavioral Intention, & Exercise Enjoyment)

To develop measures of key cognitive constructs shown in Figure 1 (self-efficacy, behavioral intention, and exercise enjoyment), we relied on survey data provided by the students in our sample. The questions posed in the survey were informed by SCT and Simpson et al.’s work [24] and included items adapted from those used by Obarzanek and Pratt [47]. The original scale was tested for construct validity [47] to measure diet and PA among young African American girls. Due to the potentially distinctive nature of the study population in our sample, we pilot tested the revised instrument with approximately 30 students from grades 2–5 in the school district we investigated and used the results from the pilot test to revise the questionnaire for use with this population. The findings from the pilot study suggested that the children had difficulty differentiating between PA, recent exercise, leisure, and moderate to vigorous PA when asked about these concepts separately, but they did tend to recall the PA they had engaged in over the past week. As such, the terms PA and exercise are used interchangeably in the self-report questionnaire, as well as in this manuscript as is noted above.

The self-efficacy scale used in this study was derived from Obarzanek and Pratt’s study [47], and included responses to queries asking participants to rate their confidence with exercise:

“It is important for me that exercise makes me feel good”;

“I like to get feedback on how I’m going with my exercise”, and;

“I don’t feel tired at all when I move”.

Each question consists of a three-point ordinal Likert scale anchored by 1 (do not agree) to 3 (strongly agree), and responses were analyzed using confirmatory factor analysis to produce variable measures to include in the SEM model. Prior to using these factors, Spearman rank correlation tests for non-parametric ordinal scales were used to measure the degree of association among the responses students gave to these three queries. The scale was shown to have high consistency among the children in our sample (Spearman’s rank correlation coefficient = 0.94).

Two questions were used to capture a latent measure of children’s behavioral intentions relating to PA behaviors [47]. They were:

“Do you believe exercise will make your physical body condition better?” and

“Do you believe exercise will improve your physical and mind strength?”

These questions were anchored by 1 (do not agree) to 3 (strongly agree), and higher scores reflected higher levels of behavioral intention. As with the self-efficacy variable above, confirmatory factor analysis was used to produce factors to be incorporated in the SEM model. The Spearman rank correlation of this ordinal construct in our sample was 0.95, indicating high levels of correlation among responses to these questions.

The latent construct of exercise enjoyment was measured based on responses to three questions included in the survey referenced above. They were:

“Do you enjoy yourself while exercising in the class?”

“Do you enjoy yourself when you exercise out of the school or at home?”

“Do you like to practice moderate–vigorous physical activity in your free time?”

Rank order responses to these questions were captured on three-point Likert scale with endpoints ranging from “1 = never” to “3 = always. Responses to the three queries reflect a latent class measure of exercise enjoyment, and confirmatory factor analysis of the responses received was used to produce variable measures that were incorporated into the SEM model. The Spearman’s ordinal rank correlation for this construct was 0.91, indicating high levels of correlation within student responses to these questions.

#### 2.3.3. Mediating Social Factors

To examine relevant variation between the three sources of social support and PA, participants were asked to respond to three statements:

“My parent(s) help me exercise every day”;

“My school physical education teacher helps me exercise every day”;

“My friends help me exercise every day”.

The students’ possible responses were limited to three points on a Likert scale anchored by 1 = “never” to 3 = “very often”. To identify the effects of social support in a simpler and more easily interpreted way, we converted the three-point values for each of these social factors into binary variables (0 = no, representing “never”; 1 = yes, representing “very often” and “sometimes”) and included these binary values to test hypothesized relationships in the SEM model.

### 2.4. Statistical Analyses

Data management and descriptive univariate analysis took place using SAS 9.4 (SAS Institute Inc., Cary, NC, USA) [48], and invariance assumption tests and the final SEM model testing were conducted in M-plus 7.4 [49]. The model was estimated using the mean- and variance-adjusted weighted least squares (WLSMV) estimator because it optimizes estimators that may be non-normally distributed [50]. The weighted matrix in the WLSMV was less biased and more accurate than maximum likelihood parameter in estimating the factor loadings across ordinal constructs [51].

Data on the cognitive correlates were initially analyzed using confirmatory factor analysis to produce latent variable measures (e.g., factors) to test for construct and discriminant validity of the study measures. As is noted above, Spearman’s rank order correlation coefficients were used to verify associations across the individual measures of each of the three cognitive correlates (self-efficacy, behavioral intention, and exercise enjoyment). Measures of self-efficacy and behavioral intention were based on SCT [17] and previous analytic models [33,52,53].

The proposed pathways of influence on PA in the study were tested using SEM, and latent constructs were estimated from observed measures as described above. As Figure 1 suggests, we assessed the effects of the three sources of social support as mediators between predisposing cognitive factors (self-efficacy, behavioral intention, and exercise enjoyment) and children’s weekly PA engagement in the proposed hypothesized model. The latent variable constructs were standardized to have variance equal to 1 and standardized (β) coefficient estimates were obtained along with model-based standard errors. The coefficients (β) for paths with dichotomous dependent variables such as reported support from parents, PE teachers, and peers were log-transformed to produce odds ratios of the likelihood of perceived social support (e^β(log odds)^). Chi-square tests with two-sided *p* values were used, with a significance level of 0.05. However, given that the traditional Chi-square is sensitive to sample size [54], the model was further evaluated with model goodness of fit indices including Confirmatory Fit Index (CFI) at or above 0.90, and Tucker Lewis index (TLI) at or above 0.95, Root Mean Square Error of Approximation (RMSEA) less than 0.06, and a Standardized Root Mean Square Residual (SRMR) less than 0.08, all of which were deemed satisfactory for well-fitting model [55].

The total estimated effects of the cognitive correlates in the SEM are calculated by adding direct effects to indirect effects. Indirect effects are calculated by multiplying the pathway estimates associated with intervening variables in the SEM model, using M-plus software. To reduce the likelihood of bias in the model results, we also ran analyses, including control variables for gender, family type, and race, across the entire model. Of these variables, only gender achieved statistical significance in any portion of the model. As a result, we also ran the model using only the control for gender. The results flowing from both this model and the model with controls for all three demographic variables (family type, race and gender) were not significantly different than the results for a generic model without these control variables. The Akaike’s information criteria (AIC) statistic for model selection was used to test all three models—one with all three control variables, one controlling for gender, and a model without these control variables—to help us select the model with the best fit to the data. Based on the results of this test and other goodness of fit measures, we report the results from the generic model (without controls) in this article because it performed more strongly in terms of the goodness of fit measures mentioned above and revealed substantively similar results. Readers interested in reviewing the results from all three of these alternative model specifications may contact the first author of this manuscript for further information.

## 3. Results

### 3.1. Findings

The results flowing from our work suggest that the children in our sample have characteristics that appear typical of children living in at-risk urban environments. They also suggest that constructs associated with self efficacy and exercise enjoyment have stronger direct and total effects on PA than behavioral intentions. In addition, for the children in our sample, the results suggest that support from PE teachers and peers may have greater positive effect on their PA than does parental support. In the subsections that follow, we review these results in greater detail.

### 3.2. Descriptive Univariate Analysis

Table 1 displays descriptive characteristics for the children in our sample. The sample consists of 725 children, with 346 boys (47.7%) and 379 girls (52.3%), and more than half of them indicated that they had a single parent (n = 373, 51.5%). The children were also predominantly identified as African American (n = 462, 63.7%). For boys, the average total weekly minutes of PA was 286.2, while girls’ weekly average PA engagement was 213.95 min per week—about 72 min less than the boys. These statistics, along with the use of the USDA’s federal school lunch program in this school district in Ohio, suggest that our sample of children has characteristics consistent with those of children who are growing up in a predominantly minority urban community in the Midwest US.

### 3.3. Model Fit

Table 2 shows factor loadings of latent variables measuring conceptual constructs and significant correlations associated with self-efficacy (0.42, 0.51, 0.62), exercise enjoyment (−0.60, −0.71, −0.84), and behavioral intention (0.61, 0.70). The correlations across these factors all exceed 0.40, a correlation threshold identified as “moderate” by Fabrigar and his colleagues’ study for factor analysis [55].

**Figure 2:**
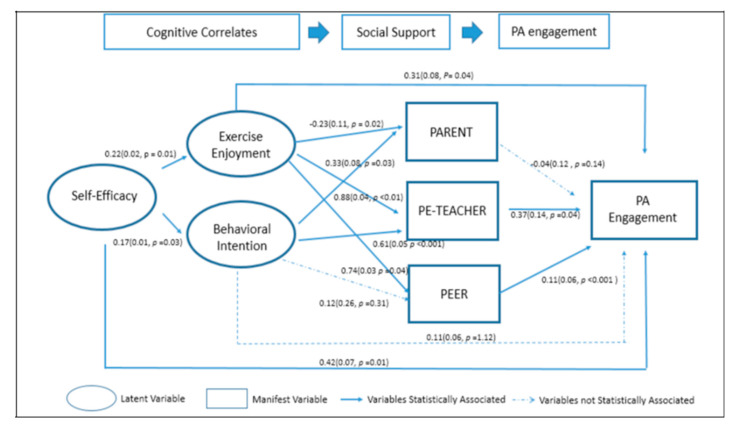
The estimated path coefficients of the hypothesized model. Standardized coefficients (β), standard error (SE) and *p*-value are included in the graph, β (SE, *p*-value). Model fit indicators: *χ*2 (725, *df*: 21) = 54.03 (*p* < 0.01), Root Mean Square Error of Approximation (RMSEA): 0.04, Standardized Root Mean Square Residual (SRMR): 0.04, Confirmatory Fit Index (CFI): 0.89, Tucker Lewis index (TLI): 0.95 Explained variance of the model: 45.2% based on M-plus’ measure of explained variance.

Figure 2 shows the estimated path coefficients of the hypothesized model for children’s total weekly PA minutes, as well as model fit statistics, including a RMSEA of 0.04 (95% CI: 0.03–0.06) and a SRMR of 0.06. The overall model fit of the model was acceptable, despite a *χ*2 (725, df: 21) = 54.03 (*p* < 0.01). The calculated Chi-square fit between the saturated model (population model) and the current model resulted in moderate CFI (0.89), suggesting moderate fit as the CFI value is slightly below the 0.90 cutoff. However, the value of TLI is 0.95 and falls within the statistically recommended ranges, indicating goodness of fit [54]. Overall, the model accounts for 45.2% variance of weekly PA engagement.

### 3.4. Direct Effects of Key Cognitive Correlates.

As is noted above, Figure 2 shows the estimated path coefficients of the hypothesized model. The model reveals statistically significant direct associations between two of the cognitive constructs—self-efficacy (β = 0.42, *p* = 0.01) and exercise enjoyment (β = 0.31, *p* = 0.04)—and reported weekly PA. These standardized estimates can be interpreted as a one unit increase in self-efficacy having a 0.42 min increase in weekly PA, and a one unit increase in exercise enjoyment having a 0.31 min increase in weekly PA. The behavioral intention construct did not attain a statistically significant relationship with reported PA (β = 0.11, *p* = 0.21).

The model results also reveal statistically significant relationships between self-efficacy and both exercise enjoyment and behavioral intention. A one-unit increase in the self-efficacy construct was associated with a 0.22 unit increase in exercise enjoyment (*p* = 0.01) and a 0.17 increase in behavioral intention (*p* = 0.03), respectively. This finding suggests that feelings of self-efficacy may lead children to both enjoy their exercise activities and feel greater confidence in their ability to achieve intended results, both of which may contribute toward PA indirectly through specific sources of social support.

The results also suggest relationships between exercise enjoyment and behavioral intentions and children’s perceptions of social support from parents, PE teachers and peers. As the information provided in Table 3 suggests, exercise enjoyment is associated with 2.1 times greater odds of peer support (e^0.74^ = 2.1, *p* < 0.05), interpreted as a one unit increase in exercise enjoyment makes it about two times more likely that a child will perceive strong peer support for PA activities. Similarly, exercise enjoyment makes it about 2.4 times more likely that the child will perceive PE teacher support for PA (e^0.88^ = 2.4, *p* < 0.01). By contrast, exercise enjoyment was associated with a 23% decrease in the odds of parental support, as a one unit increase in exercise enjoyment makes it about 23% less likely that the student will report strong parental support for PA (e^−0.23^ = 0.79, *p* = 0.02). Behavioral intentions were statistically associated with 1.8 times greater odds of perceived PE teacher support (e^0.61^ = 1.8, *p* < 0.001) and 1.4 times greater odds of reported parental support (e^0.33^ = 1.4, *p* < 0.05). By contrast, behavioral intentions yield 1.2 times greater odds of reported peer support (e^0.12^ = 1.2, *p* = 0.31), but this result is not statistically significant.

### 3.5. Total and Indirect Effects of Cognitive Correlates

In Table 4, we show total effects of each of the cognitive correlates, and they result from the summation of direct and indirect effects. According to the results of the tested model, self-efficacy had indirect effects on PA mediated by behavioral intention and exercise enjoyment, and–further–by sources of social support (0.36). However, about 54% of the total effect of the self-efficacy construct is revealed to be through direct influence on children’s PA (0.42), while about 46% (0.36) of self-efficacy’s impact on PA is indirect and operates through impacts of self-efficacy on both exercise enjoyment and behavioral intention.

Exercise enjoyment has both direct and indirect effects on PA, the latter of which operates through perceived support from PE teachers, peers, and parents (totaling 0.35). Of the total effect of exercise enjoyment, the magnitude of the teacher’s indirect effect (0.21) is larger than the other two sources of social support (peer = 0.12 and parental support = 0.02, the latter of which is not statistically significant). Overall, about 47% of the total effect of exercise enjoyment is direct (0.31) and about 53% (0.35) appears to reflect indirect pathways of influence on PA. Of the indirect effects of exercise enjoyment, PE teacher support (60%) outperforms the other two sources of support (34% peer and 6% parent, the latter of which is not statistically significant) with regard to impacts on reported PA.

The model results also suggest that while behavioral intentions fail to have statistically significant direct effects on PA, they do have statistically significant impacts on perceptions of support from parents and PE teachers. However, perceptions of social support from parents do not appear to be a statistically significant, and behavioral intentions do not have statistically significant effects on perceived support from peers. Overall, therefore, behavioral intentions’ indirect influence on PA results from its influence on reported support from PE teachers, which has a total estimated effect of 0.05—all of which is tied to perceived support from PE teachers.

Overall, the results described above and displayed in Figure 2 suggest that self-efficacy and exercise enjoyment have greater total effects on PA for the children in our sample than do behavioral intentions.

### 3.6. Sources of Social Support for PA

Our results also provide estimates of the effects of various sources of perceived social support on reported PA. Two of these mediating factors, support from PE teachers (β = 0.37, *p* < 0.05) and support from peers (β = 0.11, *p* < 0.01), have statistically significant direct associations with reported weekly PA. By contrast, children’s perceptions of parental support fail to achieve statistical significance in its effects on PA in the model. In addition, its sign is negative, suggesting a possible negative effect of perceived parental support on PA (β = −0.04, *p* > 0.14). Overall, these results suggest that support from PE teachers and peers have positive effects on PA among the at-risk children in our sample.

## 4. Discussion

Our findings re-enforce past findings in some respects and add to them in others. They also yield implications for the design and implementation of interventions to increase PA among at-risk urban children. Our findings also have limitations that should be recognized and may productively inform further research. The discussion below provides further insights in each of these areas.

### 4.1. Contributions to Knowledge

Our findings lend further empirical support for the value of cognitive constructs drawn from SCT and other theoretical perspectives in explaining variations in PA [15,39]. In particular, they suggest that self-efficacy and exercise enjoyment may influence the extent to which at-risk urban children engage in PA. We did not, however, find statistically significant relationships between behavioral intention and PA, except insofar as behavioral intention may indirectly influence PA through a positive relationship with student perceptions of PE teacher support. These findings are broadly consistent with past studies suggesting that SCT-based constructs influence PA [19,20,33], but expand upon those findings by suggesting that the influences of SCT concepts are operative among at-risk children and exert influences indirectly through children’s perceptions of external sources of social support as well as directly on PA. In this regard, our findings support the idea that SCT constructs influence the behavior of at-risk urban children, in spite of earlier suggestions that the influence of SCT-based variables may increase as people mature and grow older [18].

Our study target population was relatively young and still appears to have been influenced by SCT-based variables. In part, because our targeted study population was young, we might expect their PA to be influenced by external sources of social support [54]. In this regard, our results broadly echo past findings suggesting that social support from parents, peers, and/or PE teachers may have positive impacts on PA [21,36,56,57]. However, our findings also suggest that influential pathways of social support may operate in the broader context of the cognitive orientations of the children involved. Children with stronger senses of self-efficacy appear more likely to be characterized by goal-oriented behavioral intentions and exercise enjoyment. In addition, children who enjoy exercise appear more likely to report encouragement and support from their PE teachers, peers and parents. Among the sources of social support for the children in our low-income urban sample, PE teachers appear to have the strongest influence on PA, followed by peers. External support from parents, by contrast, does not achieve a statistically influence on PA among the children in our sample. Overall, our findings of statistically significant associations between both PE teacher and peer support and children’s PA suggest that these two sources of social support may be particularly important for the at-risk urban children in our sample.

### 4.2. Informing Practice, Policy, and Interventions to Increase PA

Our findings, as summarized above, yield insights that may be used to guide interventions to encourage PA among at-risk urban children. They provide insights suggesting that efforts to enhance feelings of self-efficacy and exercise enjoyment on the part of at-risk urban children may be helpful in encouraging PA and may also appropriately accompany efforts to use sources of social support in behavioral change strategies for this target population.

Broadly, our findings suggest that there may be value in interventions to support PA by building feelings of self-efficacy and the enjoyment of exercise among at-risk urban children. To the extent that feelings of self-efficacy among children are influenced by their home situations, this finding suggests that educational outreach and the development of other mechansims to imbue young children with a sense that they may effectively influence the environment around them may be helpful in encouraging PA. While educational outreach to parents and care-givers in at-risk urban areas would be consistent with this suggestion, educational efforts of this kind that are directed at day care centers and schools for young childern might also be appropriate.

Outreach to educators might also include efforts to emphasize the importance of developing opportunities for at-risk children to enjoy exercise activities. While our previous work [36] suggests that motivational strategies used by PE teachers influence PA, the findings presented above suggest that one key motivational strategy to be utilized in at-risk urban schools may be to find ways to increase the enjoyment of exercise by students, both in the classroom and, to the extent possible, beyond it as well.

Our findings also suggest that behavioral change strategies to support PA may be productively designed to encourage PE teachers and peers to positively influence PA among at-risk urban children. While our previous study [36] suggested that motivational strategies used in PE curricula and classroom delivery might be focused productively toward this end, the findings presented here re-enforce this recommendation and suggest that these efforts may also benefit from a focus on ways in which peers, as well as PE teachers, may be enlisted to contribute to encouraging PA among children in at-risk urban environments.

### 4.3. Limitations and Further Research

While we believe the insights presented above are useful and should be considered by urban school districts and communities as possible avenues to enhance childhood PA practices, we also recognize that there are limitations associated with this work. Virtually all studies have limitations, and we believe that at least three limitations are appropriate to acknowledge here, specifically in the hope that they may help guide further research in this area.

Firstly, our findings are based on one set of surveys completed by an at-risk group of children in one midwestern city in the US. While Akron Ohio and the results presented may be typical of urban at-risk children in the US, replication and/or verification of these findings in other urban environments would be appropriate, and could build greater confidence regarding the findings presented above.

Secondly, while our SEM model is used in an effort to recognize complexities associated with factors that influence PA among at-risk urban children, it may not include information on all of the relevant factors that influence PA. Indeed, as is noted above, our model is reported to explain less than half of the variance in PA reported by the children in our sample, and this means that other factors may be important to include in future modeling efforts. For example, the model was constructed in ways that did not account for variations in parent socio-demographics such as education, marital status, income, and living circumstances. In addition, while our modeling efforts address perceived support from parents, peers, and PE teachers, they do not address other sources of social support such as siblings and other adult mentors. Inclusion of information on these and other factors in future modeling efforts might further inform the influence of cognitive correlates and sources of social support on PA among at-risk urban children.

In addition, our findings might be further clarified if we included information about not only the families involved, but also the physical environments in which they live. Parent(s) who live in urban communities may be more attentive to environmental safety barriers and/or have less access to safe sports facilities [1,58]. This reality may lead parents to take actions to prevent their children’s PA engagement for safety purposes. Measures on neighborhood safety and neighborhood environment were not included in our SEM model. Future modeling efforts may be improved by adding environmental factors, which may yield further insights regarding our findings on parental support and could potentially increase the explained variance of children’s PA in expanded models.

Thirdly, Bandura emphasizes that SCT is “embedded in a network of reciprocal causation” [16] (p. 508). Our SEM model, however, envisions a uni-directional flow of causal influence, which flows from self-efficacy to other cognitive correlates, and then to child perceptions of support from external sources to reported PA. While this model is informative because it recognizes both the potential for cognitive correlates to influence sources of social support and the important influences of sources of support for children, other potential causal sequences drawing from SCT and other cognitive orientations might also be modeled in ways that produce useful insights [40]. For this reason, it would seem appropriate to test hypotheses relating to SCT using statistical designs that seek to better account for reciprocally-structured relationships. While this may be difficult to do definitively, efforts of this kind are nevertheless appropriate because they recognize the multiple complexities associated with the factors driving PA among at-risk urban children.

## 5. Conclusions

Children in at-risk urban environments face disproportionate risks of obesity and other conditions that may be alleviated by interventions to increase childhood PA [59,60]. The factors influencing the extent to which these children engage in PA are multi-faceted and complex. In this study, we have sought to engage in this complex set of relationships by using an SEM to build on insights flowing from SCT to analyze both cognitive orientations of the children involved and their sources of social support for PA. In so doing, we have produced evidence that feelings of self-efficacy and exercise enjoyment influence PA for this particular audience, while also illuminating potentially valuable roles that social support from PE teachers and peers may play in enhancing PA engagement. Our hope is that the results and discussion presented above may encourage further thinking about interventions to increase PA in this population, while also providing insights to inform further research in this area.

## Figures and Tables

**Table 1:** Descriptive statistics: children’s demographics and indicators of social support.

	Sample			Weekly PA	
Category	n	%	Mean (min)	Standard Deviation (min)	
*** Sex***					
Boys	346	47.7	286.2	131	(272.3–300.0)
Girls	379	52.3	214	94.2	(204.4–223.5)
*** Family types***					
Single parents	373	51.5		120.4	(249.8–274.3)
Both parents	281	38.8		121.6	(225.5–254.1)
Other relatives without parents	37	5.1		65.1	(174.7–218.1)
Others	34	4.7		99.4	(192.3–261.6)
*** Race***					
Black	462	63.7		123.9	(241.6–264.3)
White	155	21.4		119.8	(236.4–274.4)
Asian	22	3		79.7	(147.3–226.6)
Latino/Hispanic	39	5.4		94.4	(186.9–248.2)
Others	47	6.5		63.9	(181.6–267.5)
*** Parental support***					
Very Often	276	38.1		90	(211.2–232.6)
Sometimes	242	33.4		121	(247.2–277.9)
Never	207	28.6		141.8	(247.9–286.7)
*** Peer support***					
Very Often	200	27.6		159.6	(278.3–322.8)
Sometimes	218	30.1		97.6	(250.9–277.0)
Never	307	42.3		78	(194.7–212.2)
*** Teacher support***					
Very Often	153	21.1		155.7	(333.1–382.9)
Sometime	249	34.3		93.8	(245.8–269.2)
Never	323	44.6		65.3	(182.4–196.7)
*** Grade (Proxy to Age)***					
2nd	181	24.97		113.19	(240.1–273.3)
3rd	185	25.52		118.54	(222.7–257.0)
4th	159	21.93		112.66	(233.9–269.2)
5th	200	27.59		128.57	(228.5–264.4)

**Table 2:** Correlation matrix of three latent constructs.

*Latent Construct*	Factor 1		Factor 2		Factor 3		Spearman Correlation
Underlying Construct Structures							
***self-efficacy***							
It is important for me that exercise makes me feel good.		***0.62***					0.94
I like to get feedback on how I’m going with my exercise.		***0.51***					
I don’t feel tired at all when I move.		***0.42***					
***behavioral intention***							
Do you believe exercise will make your physical body condition better?				***0.70***			0.95
Do you believe exercise will improve your physical and mind strength?				***0.61***			
***exercise enjoyment***							
Do you enjoy yourself while exercising in the class?						***−0.84***	0.91
Do you enjoy yourself when you exercise out of the school or at home?						***−0.71***	
Do you like to practice moderate-vigorous physical activity in your free time?						***−0.60***	

**Table 3:** Odds ratios for relationship between cognitive correlates and sources of social support.

Sources of Social Support									
	Parent			PE Teacher			Peer		
*Cognitive Correlates*	β	OR	*p*-Value	β	OR	*p*-Value	β	OR	*p*-Value
Exercise enjoyment	−0.23	0.79	0.02	0.88	2.4	<0.01	0.74	2.1	0.04
Behavioral intention	0.33	1.4	0.03	0.61	1.8	<0.001	0.12	1.2	0.31

Odds ratio (OR) represents the likelihood that each factor of social support (parent, PE teacher, peer, all of which are measured dichotomously in the model) is reported to be present “sometimes” or “very often”, based on the effect of the cognitive correlates listed.

**Table 4:** Direct, indirect and total effects of cognitive correlates on physical activity (PA): statistically significant effects (*p* < 0.05).

Construct	Direct Effect	Mediators	Indirect Effect	Total Effect
Self-efficacy-*PA*	0.42	Self-efficacy-Exercise Enjoyment–Parent Support-*PA*	0.09	0.78
		Self-efficacy-Exercise Enjoyment–PE Teacher Support-*PA*	0.11	
		Self-efficacy-Exercise Enjoyment-Peer Support-*PA*	0.07	
		Self-efficacy-Behavioral Intention–PE Teacher Support-*PA*	0.07	
		Self-efficacy-Behavioral Intention-Peer Support-*PA*	0.02	
Exercise Enjoyment-*PA*	0.31	Exercise Enjoyment-Parent Support-*PA*	0.02	0.66
		Exercise Enjoyment-PE Teacher Support-*PA*	0.21	
		Exercise Enjoyment-Peer support-*PA*	0.12	
Behavioral intention-*PA*		Behavioral intention–PE Teacher Support-*PA*	0.05	0.05
